# Early Rooming Triage: Accuracy and Demographic Factors Associated with Clinical Acuity

**DOI:** 10.5811/westjem.2021.12.53873

**Published:** 2022-02-28

**Authors:** David Y. Zhang, Bradley Shy, Nicholas Genes

**Affiliations:** * The Mount Sinai Hospital, Department of Emergency Medicine, New York, New York; † New York-Presbyterian Hospital, Columbia University Irving Medical Center, Department of Biomedical Informatics, New York, New York; ‡ Denver Health and Hospital Authority, Department of Emergency Medicine, Denver, Colorado; § University of Colorado School of Medicine, Aurora, Colorado; ¶ Ronald O. Perelman Department of Emergency Medicine, NYU Langone Health, New York, New York; || NYU Grossman School of Medicine, Department of Emergency Medicine, New York, New York

## Abstract

**Introduction:**

Early rooming triage increases patient throughput and satisfaction by rapidly assigning patients to a definitive care area, without using vital signs or detailed chart review. Despite these operational benefits, the clinical accuracy of early rooming triage is not well known. We sought to measure the accuracy of early rooming triage and uncover additional patient characteristics that can assist triage.

**Methods:**

We conducted a single-center, retrospective population study of walk-in emergency department (ED) patients presenting to the ED via an early rooming triage system, examining triage accuracy and demographic factor correlation with higher acuity ED outcomes.

**Results:**

Among all patients included from the three-year study period (N = 238,457), early rooming triage was highly sensitive (0.89) and less specific (0.61) for predicting which patients would have a severe outcome in the ED. Patients triaged to the lowest acuity area of the ED experienced severe outcomes in 4.39% of cases, while patients triaged to the highest acuity area of the ED experienced severe outcomes in 65.9% of cases. An age of greater than 43 years (odds ratio [OR] 3.48, 95% confidence interval: 3.40, 3.57) or patient’s home address farther from the ED ([OR] 2.23 to 3.08) were highly correlated with severe outcomes. Multivariable models incorporating triage team judgment were robust for predicting severe outcomes at triage, with an area under the receiver operating characteristic of 0.82.

**Conclusion:**

Early rooming workflows are appropriately sensitive for ED triage. Consideration of demographic factors, automated or otherwise, can augment ED processes to provide optimal triage.

## INTRODUCTION

### Background

Emergency department (ED) triage is a continually evolving process, changing in response to both emerging patient characteristics and improved understanding of fundamental pathophysiological processes. Prior to the development of scales such as the Emergency Severity Index (ESI), triage decisions were heterogeneous and variable. Significant inter-rater variation was observed when nurses and emergency medical technicians were tested with standardized clinical scenarios,^1^ and nurses and physicians were not able to accurately predict the need for admission at triage.^2^ In response, the five-point ESI scale was developed and validated. The ESI score has good inter-rater reliability and is predictive of hospital admission,^3^ especially at the extremes of triage scores.^4^ Additionally, the ESI score is validated in a range of specialized populations, such as pediatric patients,^5^ leading to its widespread use in ED triage.

Since the validation of these triage risk assessment tools almost 20 years ago, ED triage processes have changed to progressively improve ED throughput and patient satisfaction. In addition, disease-specific triage pathways have emerged for acute clinical presentations, such as stroke, sepsis, and acute coronary syndrome. Although these changes have improved care for critically ill patients and improved ED metrics, they concurrently demand more of triage teams, requiring greater accuracy with less initial information. Given these changes, it is essential to both validate the performance of these systems and discover patient characteristics that improve risk stratification.

### Importance

Triage workflow has since expanded beyond ESI. To improve ED front-end operations, two complementary and successful approaches have emerged: practitioner-in-triage systems, and early rooming systems (direct to bed or split flow systems).^6^ Practitioner-in-triage systems, where a physician or midlevel practitioner works in triage to expedite patient care, have demonstrated improved wait times,^7^ fewer patients waiting to be seen,^8^ and decreased length of stay.^9^ These improvements are tempered by increased physician or midlevel resources dedicated to triage. Similar benefits have been noted for early rooming strategies—patients are rapidly placed in a care area after a cursory registration process, allowing for concurrent triage, nursing, and physician assessment. Early rooming strategies have improved patient waiting times, decreased lengths of stay, and decreased rates of patients leaving without being seen.^10^,^11^ These improvements also improve patient care, as patients initially leaving without being seen nevertheless have significant rates of critical illness and need for hospitalization.^11^ Despite this progress, it is unclear whether abbreviated registration in early rooming retains the accuracy and sensitivity for critical illness, compared to traditional triage workflows.

### Goals of This Investigation

Patient demographics are continually evolving, with increasing numbers of non-urgent patients with different care requirements.^12^,^13^ The number of geriatric patients also continues to grow, with a different set of unique characteristics.^14^,^15^ A plethora of risk-stratification tools have emerged; however, they are typically either fast with poor accuracy,^16^ or require additional clinical data such as vital signs^17^,^18^ and laboratory testing.^19^–^21^ These tools are not appropriate for early rooming triage, where vital signs and testing cannot delay initial triage decisions. In response to these challenges, we now present data from a large population of walk-in patients at an urban academic ED. We assess the sensitivity and specificity of early rooming for detecting emergent outcomes and explore demographic factors available at triage that further improve triage accuracy.

**Population Health Research Capsule**

What do we already know about this issue?

*Emergency department patients are diverse, with a wide range of clinical presentations. Several different triage methods exist to manage these presentations.*

What was the research question?

*We determined the accuracy of early rooming triage, and whether use of demographic factors could improve it.*

What was the major finding of the study?

*Early rooming triage is effective and can be further improved by considering the patient’s age and ZIP code.*

How does this improve population health?

*Emergency department outcomes vary across demographic strata. Consideration of these factors will optimize resource allocation and improve patient care.*

## MATERIALS AND METHODS

### Early Rooming Triage

In our ED, early rooming triage is performed by a registration clerk and experienced nurse for all walk-in patients. After a brief discussion with the patient and registration in the electronic health record (EHR), patients are rapidly assigned to one of three care areas, specialized for treating low-, medium-, and high-acuity patients. When beds are not available in these areas, patients are moved to an available chair, hallway area, or other proximal location in the ED. This ensures the patient is rapidly available to all members of the care team, who work in parallel to manage the patient clinically. If a patient is found to need a different level of care at any time during the ED evaluation, patients are moved to higher or lower acuity areas as appropriate.

### Study Design and Setting

We conducted a retrospective population study of all walk-in ED visits at a large, academic, urban ED between January 1, 2017–December 31, 2019, examining both the accuracy of early rooming triage and demographic factors predictive of severe ED outcomes. This work was reviewed and approved by our institutional review board under protocol 16-00180.

### Selection of Participants

We included every walk-in patient triaged based on acuity. Major groups not included in the study were pediatric patients triaged based on age, and patients directly triaged to the psychiatric ED. A pilot geriatric emergency area was active during part of the study period, and patients triaged there based on age criteria were also excluded in the study. Visits with missing demographic or location data were not included in subsequent analysis.

### Methods of Measurement

We directly extracted patient demographic factors, patient behavioral factors, and disposition (including admission and death) directly from the EHR. Six outcome measures were used as surrogates for severe outcome based on their ease of extraction from the health record and their perceived correlation with patients requiring higher level of care in the ED. Since these measures have not been individually validated for this purpose, we considered them in aggregate to represent sick patients more comprehensively. Patients were considered to necessitate an operative intervention if an operative note was signed at any time during the current visit, including after admission. Sepsis alerts were triggered within the EHR based on vital sign criteria, and only counted as positive for this study if the clinician confirmed the sepsis status in a structured clinical note and order set. Targeted temperature management and intravenous (IV) epinephrine use were inferred by the presence of those signed orders during the current visit. Patients transferred to another institution or placed in the observation unit were not considered admitted in this study.

### Outcome Measures

We used a composite outcome consisting of six electronically accessible outcomes or treatments in the ED, each representing an aspect of patient care that was highly suggestive of the need for higher level of triage. Patients were considered to require higher levels of triage if they experienced any one of the following: admission to the hospital, operative intervention during the current visit, confirmed sepsis, death, IV epinephrine, or targeted temperature management. For assessing triage accuracy, patients were considered correctly triaged if they were either triaged to the medium- or high-acuity areas of the ED and subsequently developed a severe outcome, or if they were triaged to the low-acuity area of the ED and subsequently did not develop any severe outcome.

### Analysis

After EHR extraction, we performed all data processing and data analysis in SAS Studio 3.8 (SAS Institute Inc, Cary, NC). We inferred residence from ZIP code. Event frequencies, coincidence, univariate logistic, and multivariable logistic analysis were performed using their corresponding packages in SAS Studio. We performed multivariable regression by including all factors with univariate significance, assuming no interaction between individual factors.

## RESULTS

**Figure 1:**
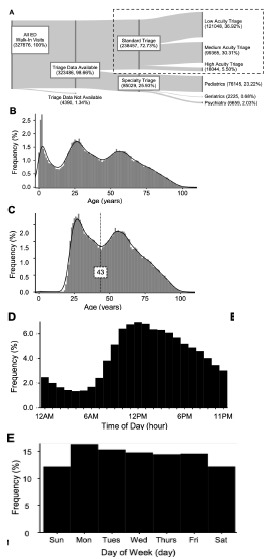
Patient inclusion and baseline demographic figures. (A) Distribution of all walk-in emergency department (ED) patients arriving during the study period, with aggregate triage processes and triage destinations. Vertical bars are proportional to the number of patients in each group. The dashed line surrounds the patients included in subsequent analysis. All percentages are expressed as a fraction of all walk-in patients. (B) Age distribution of all walk-in ED patients with local trendline. (C) Age distribution of patients included in subsequent analysis, after specialty triage has been excluded. The vertical dashed line splits the patients < 43 years of age from the patients ≥ 43 years of age. (D–E) Distribution of arrival times and arrival day for patients undergoing standard triage.

There were 327,876 patient visits to the walk-in triage area of the ED during the study period, and of these visits, triage data was available for 323,486 (98.66%). Of all visits, a subset (25.93%) was triaged to specialized pediatric, psychiatric, or geriatric areas using criteria that were not based on acuity and were not included in the study. The remaining 238,457 patients (72.73%) underwent triage based on perceived acuity to low-, medium-, and high-acuity areas of the ED (Figure 1A).

The distribution of all walk-in patients was trimodal, representing pediatric, young adult, and older adult populations (Figure 1B). Since pediatric patients were excluded from the study population, the patients who were included in the study formed a bimodal distribution with a central nadir at age 43 (Figure 1C). Based on the shape of the distribution, we separated patients into two age groups to correlate with outcomes: one with patients aged 42 or younger, and one with patients three or older. Patients arrived during the day more than twice as often as at night (Figure 1D). Additionally, there was a small increase in volume on Mondays, decreasing as the week went on (Figure 1E). Patients walking into the ED were slightly more likely to be female, and most had previously visited the same ED. Although our ED is in Manhattan, less than 60% of walk-in patients resided in Manhattan; most of the remainder resided in one of the other boroughs of New York City. Seven percent of patients either resided in ZIP codes outside the above-mentioned areas, or they had no ZIP code information, leading to an additional demographic group (Table 1).

**Figure 2:**
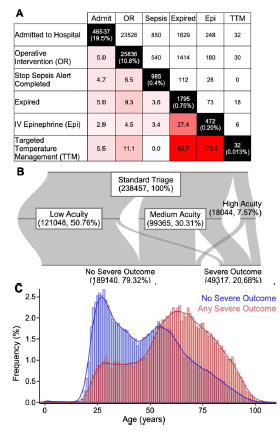
Frequency and coincidence of severe outcomes. (A) Frequencies and coincidence of the six outcomes assessed. For each outcome, the total number of patients and percentage as a fraction of all study patients are listed along the black diagonal. Above the diagonal, the number of visits with both intersecting outcomes is listed. Below the diagonal, the fold enrichment above what would be expected based on the product of their individual frequencies, the fold enrichment would be 5. Increasing red color indicates higher levels of co-incidence. (B) Proportions of patients experiencing any severe outcome, divided by initial triage area. Horizontal bars are proportional to the number of patients in each subgroup. Percentages are expressed as fraction of the study population. (C) Age distribution of patients, separated into patients experiencing any severe outcome, vs patients experiencing no severe outcomes.

We defined six “severe outcomes” that were suggestive of a patient needing higher levels of care in the ED. These outcomes were chosen to be easily accessible in the EHR, allowing for the analysis of many patient visits. The outcomes – admission to the hospital; operative intervention; confirmed sepsis; death; IV epinephrine; and targeted temperature management—occurred with varying frequency and overlap, with the most frequent being admission, operative intervention, and sepsis. Rarer outcomes had the most overlap, including death, IV epinephrine, and targeted temperature management, consistent with the care of critically ill patients (Figure 2A). Approximately one-fifth of walk-in patients experienced at least one of these outcomes, with both older patients and patients triaged to higher acuity areas of the ED predictably experiencing more severe outcomes. Of all patients triaged to the low-acuity area of the ED, only 4.40% (5322 of 121,048) experienced a severe outcome. In contrast, 65.9% (6144 of 18,044) patients triaged to the high-acuity area of the ED experienced severe outcomes. Patients triaged to the medium-acuity area of the ED were predictably between these values, with 32.3% (32,095 of 99,365) patients experiencing severe outcomes (Figures 2B–C).

All demographic factors were significantly correlated with outcomes, likely due to the large number of patients in the study. Of those, the strongest correlations were in age and area of residence: older patients and patients traveling from most outside boroughs were two to four times more likely to have severe outcomes. Patients with prior ED visits were slightly more likely to have severe outcomes, and female patients had fewer severe outcomes compared to male patients. While not a demographic factor, triage to the medium- and high- acuity areas predicted a 10-fold and 44-fold increased chance of severe outcome, respectively, compared to low-acuity triage (Table 2). The correlation between day of the week and severe outcomes was not clinically significant. Correlation between hour of arrival and severe outcomes was remarkable only for a small drop in acuity immediately before the start of the workday (Figures S1 and S2).

**Figure 3:**
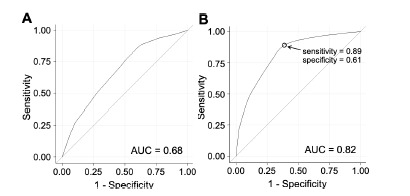
Predictive ability of multivariate models. ROC analysis for two multivariate logistic regression models to predict severe emergency department (ED) outcomes: (A) a model from Table 2 containing demographic factors alone, and (B) a model with the same demographic factors, but also including the initial triage decision made by the ED team. The circle marks the performance of the triage team alone, in a location with high sensitivity and lower specificity. *AUC*, area under the curve; *ROC*, receiver operating characteristic; *ED*, emergency department.

When used as a multivariable predictive model, demographic factors alone (Figure 3A) had reasonable predictive ability for patients with at least one severe outcome, which was greatly enhanced by adding triage team decision to the model. The measured performance of the triage team alone was similar to a single point on the combined multivariable model (Figure 3B and S3).

## DISCUSSION

In this study, we show that early rooming triage retains a high degree of sensitivity for severe outcomes, at the expense of lower specificity. This is entirely appropriate for a triage system, where inappropriate triage to a lower acuity area has the potential to cause more harm than inappropriate triage to a high-acuity area. We also demonstrate that age and patient’s location of residence are highly correlated with severe outcomes, with other demographic factors reaching statistical (but not clinical) significance. Direct extraction of these demographic and clinical fields from the EHR virtually eliminates selection and recall bias from our retrospective study design, additionally allowing for the inclusion of many patients.

This work challenges several ED stereotypes. The high-frequent visitor or “ED super-user” (a patient with recent or numerous ED visits) has been shown to have lower acuity and lower likelihood of admission compared to patients presenting to the ED for the first time.^22^ In our multivariable regression, we observed no clinically significant difference between patients with a previous visit and patients without a previous visit. We did, however, observe considerable variation in the time since last ED visit (Figure S4), raising the possibility that various binning and subgroup analyses may reveal alternate trends. Challenging another stereotype – that patients who wait until Monday to present to the ED have lower acuity compared to patients presenting over the weekend^23^ – we observed no differences between severity and day of presentation, which suggests that either sick and critically ill patients also wait until Monday to present to the ED or the rise in ED census on Mondays is due to other factors.

It is reassuring that the performance of our triage team, at a single level of sensitivity and specificity, was not significantly worse than a multivariable model including additional demographic factors. This suggests that the triage team is already considering the same or other redundant factors in the triage process. One situation where demographic factors could further enhance triage would be if we wanted to shift the sensitivity and specificity of triage to an alternate point. A higher sensitivity (but lower specificity) approach could be used when high-acuity areas of the ED were less busy. Conversely, a higher specificity (but lower sensitivity) approach could be used at times when high-acuity areas of the ED were busier. We present both human- and computer-directed triage approaches in (Figure S5).

## LIMITATIONS

This study is limited by its single-center design. It is likely that there are demographic factors specific to other EDs that are uniquely correlated with acuity. We recommend site-specific investigation of prognostic demographic factors before their use in triage. Additionally, this study included only walk-in patients in our ED, as ambulance arrivals underwent a different triage process. The risk stratification and demographic variation of ambulance arrivals is a related and interesting topic that we hope will be addressed in future work.

The breadth of outcome measures included in this study was limited by the accessible data within the EHR. While this approach allowed for the streamlined analysis of a very large number of visits, certain emergent interventions such as blood transfusion or naloxone administration were not included. We look forward to more detailed and nuanced data as our chart extraction techniques improve.

Lastly, we found a surprisingly low number of patients with confirmed sepsis in our study population. This is likely due to clinical workflow changes within the health record, or limitations in our data extraction. We have included these limited patients in our aggregate outcome measure; however, we have likely underestimated the true prevalence of confirmed sepsis in our patient population.

## CONCLUSION

Early rooming triage has previously established benefits for patient throughput and satisfaction. In this study, we demonstrate that early rooming triage nevertheless retains high sensitivity for detecting critically ill patients, despite the lack of vital signs or chart review in triage. Based on our data, we hope that ED teams will be more confident when using early rooming triage to improve ED workflow. Future studies should focus on implementation of combined clinical and computational triage processes, with the opportunity to dynamically alter triage criteria to match ED patient load.

## Supplementary Information



## Figures and Tables

**Table 1:** Selected demographic characteristics of study population.

Demographic	N	%
Gender		
Male	101,718	42.7
Female	136,739	57.3
Age		
< 43 years	93,968	39.4
≥ 43 years	144,489	60.6
Residence*		
Manhattan	147,968	62.1
Bronx	38,100	16.0
Queens	16,747	7.0
Brooklyn	16,334	6.9
Staten Island	1,815	0.8
Other/unknown	17,493	7.3
Previous ED visit		
No	70,866	29.7
Yes	167,591	70.3

* inferred from ZIP code of address.

*ED*, emergency department.

**Table 2:** Correlation between demographic factors and severe outcomes.

Demographic (comparison group)	Univariate regression		Multivariable regression	

	OR	95% CI	OR	95% CI
Gender (vs male)				
Female	0.74	(0.72 – 0.75)	0.81	(0.8 – 0.83)
Age (vs <43 years)				
≥ 43 years	3.48	(3.40 – 3.57)	3.44	(3.36 – 3.53)
Residence (vs Manhattan)				
Bronx	0.85	(0.82 – 0.88)	0.94	(0.91 – 0.97)
Queens	2.23	(2.15 – 2.31)	2.19	(2.11 – 2.27)
Brooklyn	2.32	(2.24 – 2.41)	2.38	(2.29 – 2.47)
Staten Island	3.08	(2.80 – 3.39)	2.93	(2.65 – 3.23)
Other/unknown	2.34	(2.26 – 2.42)	2.42	(2.33 – 2.51)
Previous ED visit (vs no prior)				
Yes	0.90	(0.88 – 0.92)	1.03	(1.01 – 1.05)
ED triage location (vs low acuity)*				
Medium acuity	10.37	(10.06 – 10.70)		
High acuity	42.11	(40.41 – 43.89)		

* Not included in multivariable regression

*OR*, odds ratio; *CI*, confidence interval; *ED*, emergency department.
