# Supplementary material for: Early Rooming Triage: Accuracy and Demographic Factors Associated with Clinical Acuity
Source: West J Emerg Med. 2022 Feb 28;23(2):145–51. doi: 10.5811/westjem.2021.12.53873 (PMC8967449; doi:10.5811/westjem.2021.12.53873)
Supplement: Supplementary file 1 [file wjem-23-145-s001.pdf]

Figure 1: Patient Inclusion and Baseline Demographic Factors

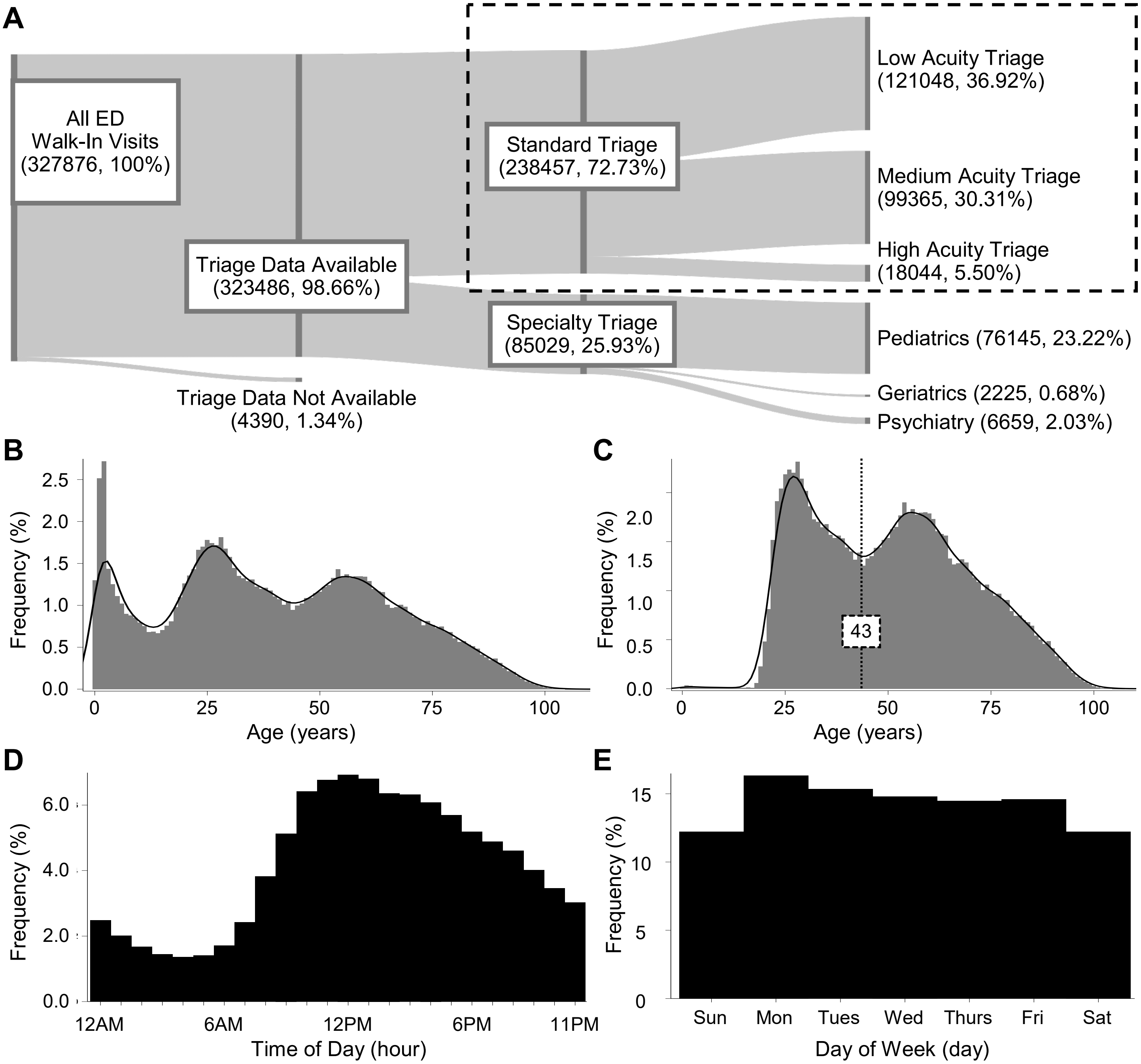

Figure 1: Patient Inclusion and Baseline Demographic Factors. (A) Distribution of all walk-in ED patients arriving during the study period, with aggregate triage processes and triage destinations. Vertical bars are proportional to the number of patients in each group. The dashed line surrounds the patients included in subsequent analysis. All percentages are expressed as a fraction of all walk-in patients. (B) Age distribution of all walk-in ED patients with local trendline. (C) Age distribution of patients included in subsequent analysis, after specialty triage has been excluded. The vertical dashed line splits the patients less than 43 years of age from the patients greater than or equal to 43 years of age. (D-E) Distribution of arrival times and arrival day for patients undergoing standard triage.

Figure 2: Frequency and Coincidence of Severe Outcomes

A

	Admit	OR	Sepsis	Expired	Epi	TTM
Admitted to Hospital	46537 (19.5%)	23528	850	1629	248	32
Operative Intervention (OR)	5.0	25836 (10.8%)	540	1414	180	30
Stop Sepsis Alert Completed	4.7	6.5	985 (0.4%)	112	28	0
Expired	5.0	9.3	3.6	1795 (0.75%)	73	18
IV Epinephrine (Epi)	2.9	4.5	3.4	27.4	472 (0.20%)	6
Targeted Temperature Management (TTM)	5.5	11.1	0.0	99.7	173.5	32 (0.013%)

B

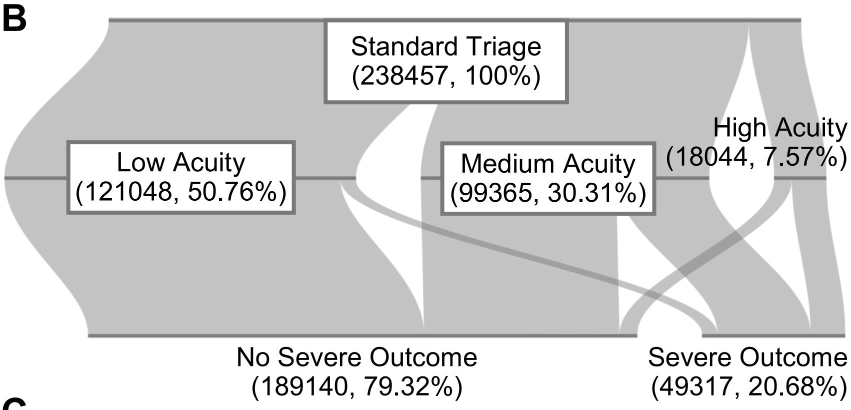

C

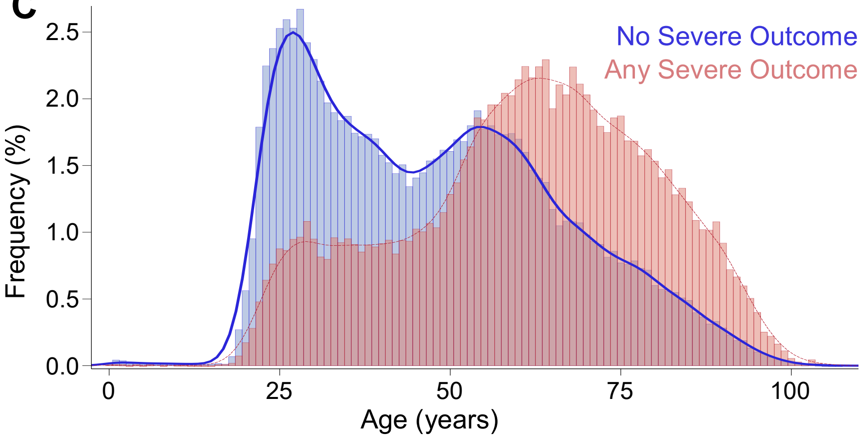

Figure 2: Frequency and Coincidence of Severe Outcomes. (A) Frequencies and coincidence of the six outcomes assessed. For each outcome, the total number of patients and percentage as a fraction of all study patients are listed along the black diagonal. Above the diagonal, the number of visits with both intersecting outcomes is listed. Below the diagonal, the fold enrichment above what would be expected by chance is listed. For example, if two outcomes occurred five times more frequently than what would be expected based on the product of their individual frequencies, the fold enrichment would be 5. Increasing red color indicates higher levels of co-incidence. (B) Proportions of patients experiencing any severe outcome, divided by initial triage area. Horizontal bars are proportional to the number of patients in each subgroup. Percentages are expressed as fraction of the study population. (C) Age distribution of patients, separated into patients experiencing any severe outcome, versus patients experiencing no severe outcomes.

Figure 3: Predictive Ability of Multivariate Models

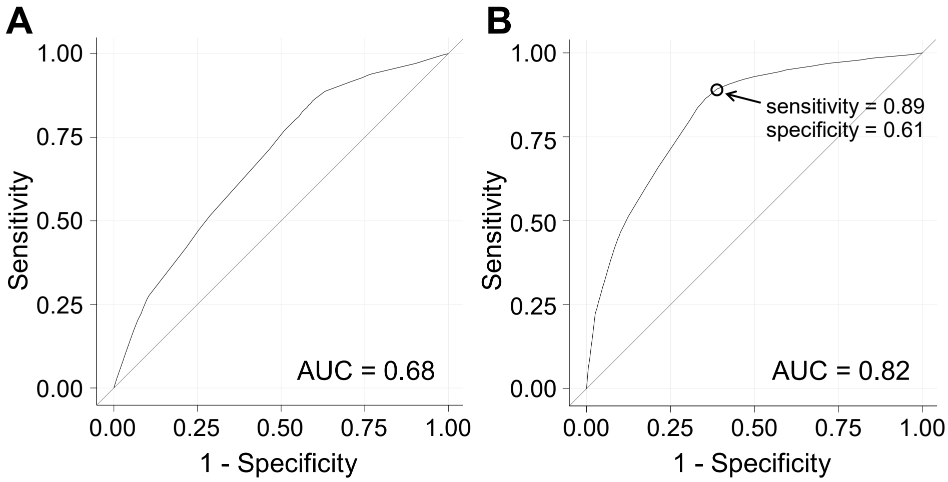

Figure 3: Predictive Ability of Multivariate Models. ROC analysis for two multivariate logistic regression models to predict severe ED outcomes: (A) a model from Table 2 containing demographic factors alone, and (B) a model with the same demographic factors, but also including the initial triage decision made by the ED team. The circle marks the performance of the triage team alone, in a location with high sensitivity and lower specificity.

Supplementary Figure 1: Correlation of Arrival Hour and Severe Outcomes

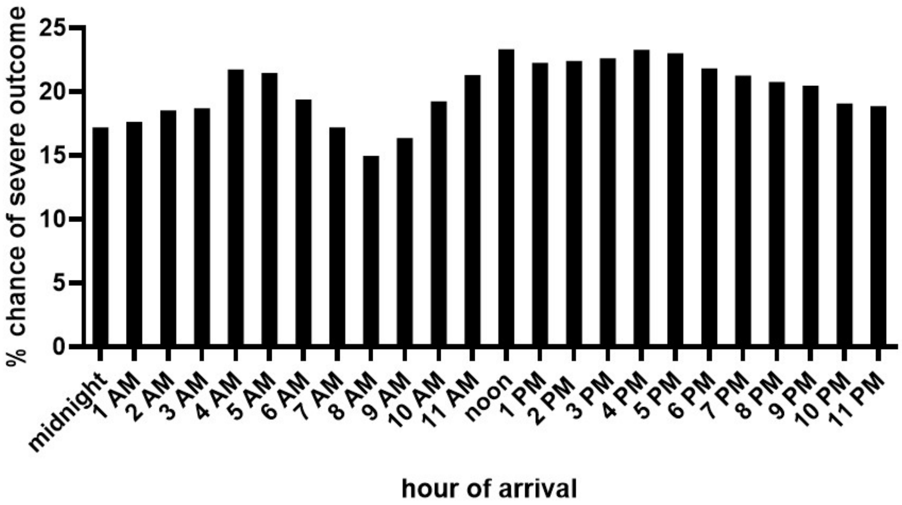

Frequency of severe outcomes by arrival time, binned by hour of arrival.

Supplementary Figure 2: Correlation of Arrival Day and Severe Outcomes

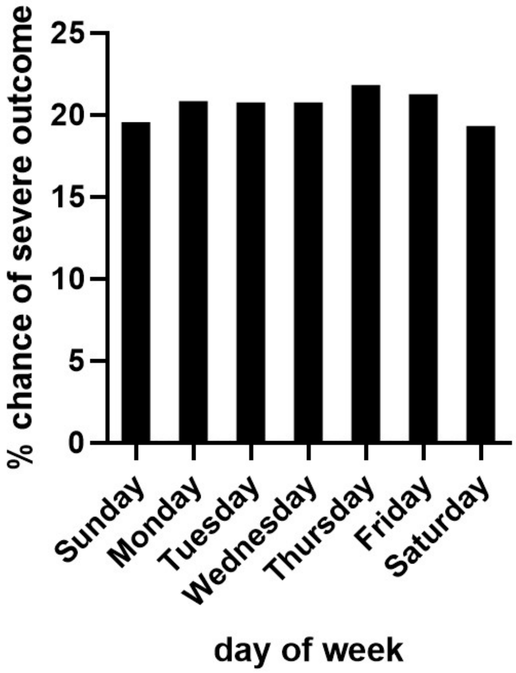

Frequency of severe outcomes by arrival day.

Supplementary Figure 3: Predictive Value of Nursing Triage Alone for Determining Severe Outcomes

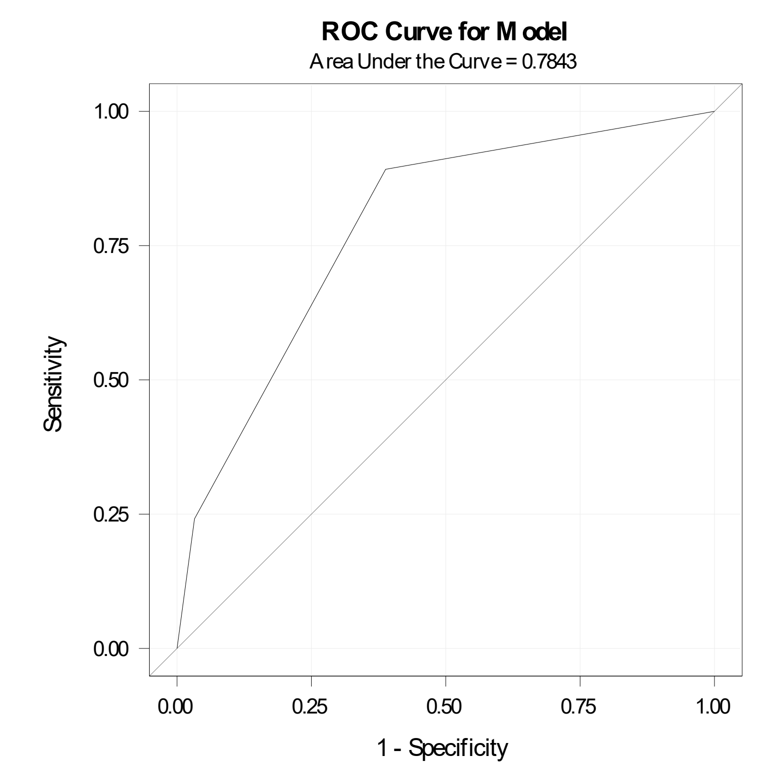

Receiver operating curve for univariate model consisting only of the existing triage decisions (triage to low, medium, and high acuity areas of the emergency room).

Supplementary Figure 4: Days since Last ED Visit in all Patients with Prior ED Visits

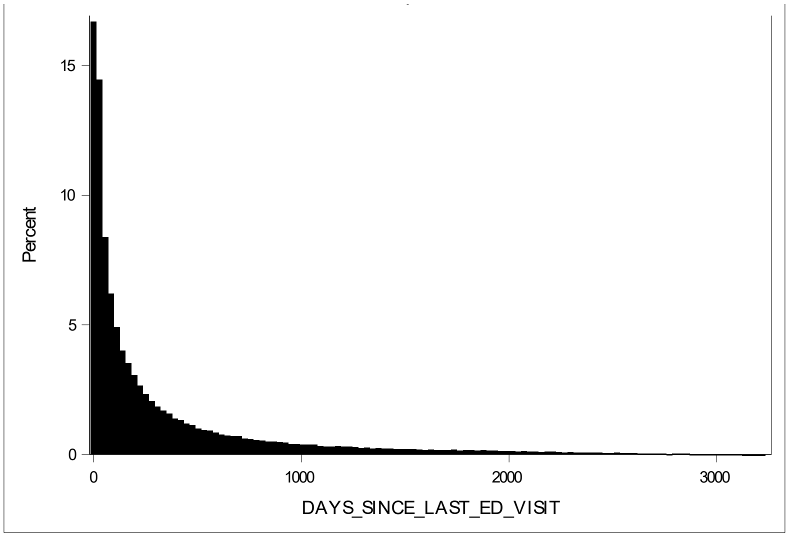

Distribution of days since last visit, among all patients with at least one prior ED visit. Each bar represents a span of one month, with considerable variation in the number of months since the prior ED visit.

Supplementary Figure 5: Approaches to Combine Demographic Data and Clinical Judgement in ED Triage

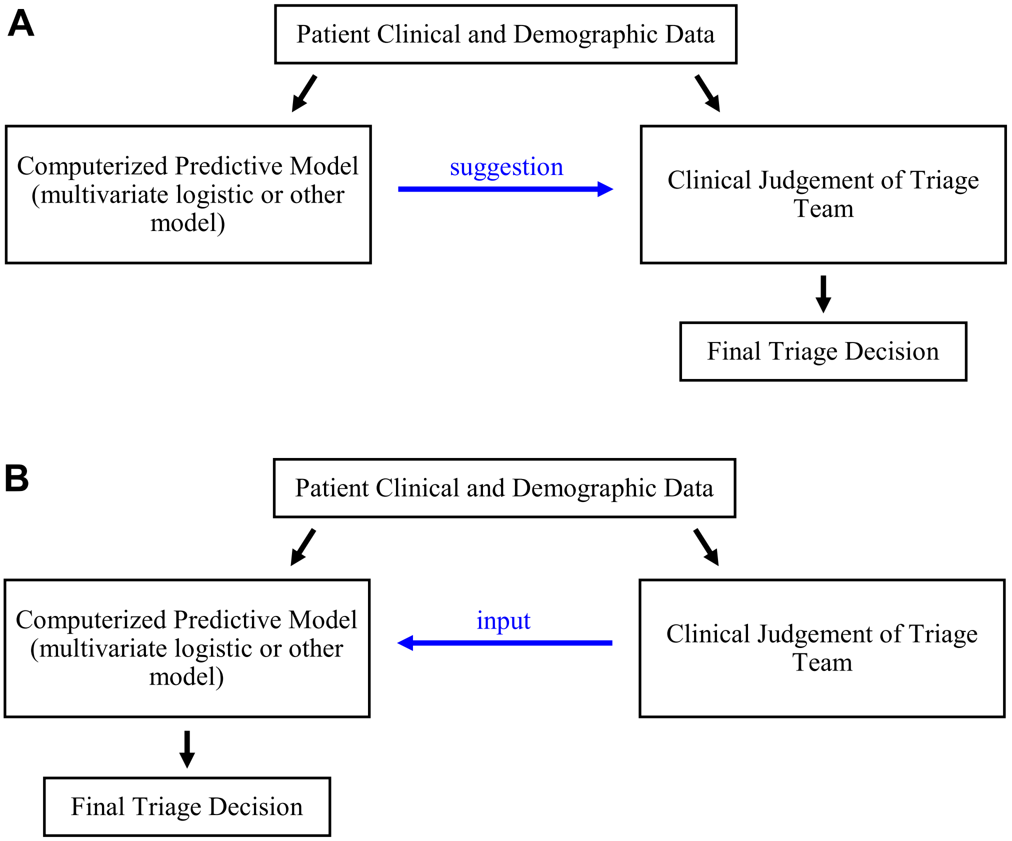

Two complimentary methods to combine demographic factors and clinical judgement in ED triage. (A) A human-led system, where a computerized predictive system offers a suggestion to a triage team, which subsequently makes the final triage decision. (B) A computer-led system, where the clinical judgement of the triage team is used as input for a predictive model, which subsequently outputs a triage decision.

Supplementary Figure 6: Association of ESI with Severe Outcome, and Collinearity Between ESI and Triage Location

Correlation of ESI and Severe Outcome

ESI	No Severe Outcome (n)	Severe Outcome (n)	Severe Outcome (%)
1	467	1526	76.6%
2	33397	23380	41.2%
3	105767	22983	17.9%
4	43011	844	1.9%
5	5106	26	0.5%
Not Recorded	1392	558	28.6%

Collinearity between ESI and Nursing Triage Location

ESI	Low Acuity Area	Medium Acuity Area	High Acuity Area
1	3	55	1935
2	3535	38876	14366
3	68549	58829	1372
4	43161	681	13
5	5095	37	0
Not Recorded	705	887	358

(darker cells represent higher numbers within a row)
